# The Benefit of Non Contrast-Enhanced Magnetic Resonance Angiography for Predicting Vascular Access Surgery Outcome: A Computer Model Perspective

**DOI:** 10.1371/journal.pone.0053615

**Published:** 2013-02-04

**Authors:** Maarten A. G. Merkx, Wouter Huberts, E. Mariëlle H. Bosboom, Aron S. Bode, Javier Oliván Bescós, Jan H. M. Tordoir, Marcel Breeuwer, Frans N. van de Vosse

**Affiliations:** 1 Department of Biomedical Engineering, Maastricht University Medical Center, Maastricht, The Netherlands; 2 Department of Biomedical Engineering, Eindhoven University of Technology, Eindhoven, The Netherlands; 3 Department of Vascular Surgery, Maastricht University Medical Center, Maastricht, The Netherlands; 4 Department of Interventional X-Ray, Philips Healthcare, Best, The Netherlands; 5 Department of MR Clinical Science, Philips Healthcare, Best, The Netherlands; University of Louisville, United States of America

## Abstract

**Introduction:**

Vascular access (VA) surgery, a prerequisite for hemodialysis treatment of end-stage renal-disease (ESRD) patients, is hampered by complication rates, which are frequently related to flow enhancement. To assist in VA surgery planning, a patient-specific computer model for postoperative flow enhancement was developed. The purpose of this study is to assess the benefit of non contrast-enhanced magnetic resonance angiography (NCE-MRA) data as patient-specific geometrical input for the model-based prediction of surgery outcome.

**Methods:**

25 ESRD patients were included in this study. All patients received a NCE-MRA examination of the upper extremity blood vessels in addition to routine ultrasound (US). Local arterial radii were assessed from NCE-MRA and converted to model input using a linear fit per artery. Venous radii were determined with US. The effect of radius measurement uncertainty on model predictions was accounted for by performing Monte-Carlo simulations. The resulting flow prediction interval of the computer model was compared with the postoperative flow obtained from US. Patients with no overlap between model-based prediction and postoperative measurement were further analyzed to determine whether an increase in geometrical detail improved computer model prediction.

**Results:**

Overlap between postoperative flows and model-based predictions was obtained for 71% of patients. Detailed inspection of non-overlapping cases revealed that the geometrical details that could be assessed from NCE-MRA explained most of the differences, and moreover, upon addition of these details in the computer model the flow predictions improved.

**Conclusions:**

The results demonstrate clearly that NCE-MRA does provide valuable geometrical information for VA surgery planning. Therefore, it is recommended to use this modality, at least for patients at risk for local or global narrowing of the blood vessels as well as for patients for whom an US-based model prediction would not overlap with surgical choice, as the geometrical details are crucial for obtaining accurate flow predictions.

## Introduction

Patients with end-stage renal disease (ESRD) have an irreversible loss of kidney function, which results in hazardous accumulation of metabolic waste products in their blood. Donor transplantation or dialysis therapy are the treatment options that can replace kidney function. Approximately 75% of the patients receive dialysis therapy [1], [2] when waiting for, or instead of kidney transplantation as donor organs are scarce. Dialysis can be divided into two categories: peritoneal dialysis (11%) and hemodialysis (89%). To perform hemodialysis, a vascular access (VA) is required to connect the artificial kidney to the blood circulation. As the typical dialysis schedule requires three dialysis sessions of four hours on a weekly basis, the VA should be easily accessible for repeated cannulation. In addition, the VA should have an adequate blood supply for efficient blood filtration by the dialysis device [2]. A VA can typically be obtained by surgical connection of an artery and a vein in the arm. Due to this bypass of the peripheral bed, the blood flow through the artery increases from approximately 47 (±5.4) to 184.2 (±12.6) ml/min one day after surgery for a lower arm VA [3]. Consecutively, vascular remodeling (dilatation) occurs [4], leading to flows of approximately 561.8 (±131.3) ml/min [3]. Unfortunately, this procedure is hampered by complication rates as high as 20%–50% [5]. Depending on the exact location in the arm, these are mainly the result of too high(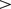
1500 ml/min [6], [7]) or too low (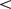
600 ml/min [8]) access flows in the upper and lower arm vascular accesses, respectively.

Predictive medicine uses computational tools in a model-based approach and aims to aid the physician in VA surgery planning by supplying additional information. Additional information for the VA surgeon could be extra details of the preoperative state [9], or a prediction of the flow after VA surgery. Recently, Bode et al. [10] demonstrated a numerical modeling framework, based on 1D wave propagation physics [11], [12], which was able to predict the direct postoperative flow resulting from VA surgery in approximately 70% of cases (n = 22) using US data as input. Direct postoperative flow is on average 60%–80% of the flow after six weeks [13] and is therefore indicative for non-maturation (insufficient access flow and vessel dilatation) and high flow related complications such as heart failure and distal arm ischemia [13], [14]. The encouraging results of Bode et al. [10] demonstrated that computational modeling of VA surgery can be considered as a valuable additional tool in the preoperative workup.

Predictive medicine by means of patient-specific computer simulation is a relatively new discipline, with a nearly inexhaustible list of challenges to be addressed. Bode et al. [10] indicated that predictions of VA surgery outcome might benefit from an increase in geometrical details, especially from identification of structures such as stenoses, which could be extracted from magnetic resonance angiography (MRA) data. The feasibility of non contrast-enhanced MRA (NCE-MRA) acquisition was demonstrated by Bode et al. [15] for a population of VA patients, and subsequently Merkx et al. [16] have compared NCE-MRA diameters with US at standardized VA vessel mapping locations. From this diameter comparison study it was concluded that arterial diameters correlate (bias 9%), while venous diameters substantially differ between NCE-MRA and US (bias 38%). Given these results, the NCE-MRA protocol of Bode et al. [15] could be used to provide additional details of the arterial geometry, and semi-quantitative information regarding the venous geometry.

Encouraged by the prior work in this field [10], [15], which claimed NCE-MRA might provide valuable geometric details for VA surgery planning, the aim of this study is to investigate this statement by applying a computer model of the blood circulation. The advantage of model-based assessment is that the influencing factors, and their interactions are combined by using physical laws. Furthermore, we are able to address the uncertainties in output values resulting from clinical measurements. The focus in this article will be on the effect that the geometric input parameters, obtained by NCE-MRA, have on the arm inflow prediction. The uncertainty in the predictions due to the geometrical measurement uncertainty was taken into account by performing a Monte-Carlo study, similar to the study of Huberts et al. [12]. To validate the approach, the patient-specific flow prediction was compared with clinical measurements after surgery. Initially, all blood vessels were assumed to be linearly tapered as in Bode et al. [10]. The cases for which differences between model predictions and postoperative measurements were found were further analyzed to determine whether an explanation could be found in the blood vessels morphology and geometry.

## Materials and Methods

**Figure 1:**
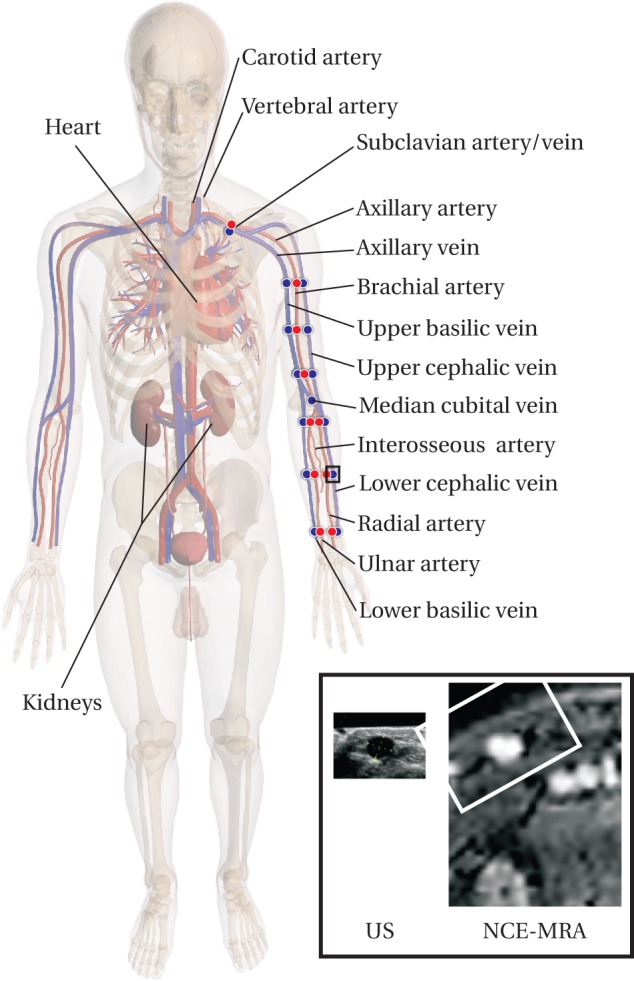
Overview of the anatomy relevant for vascular access surgery. Furthermore, the ultrasound diameter measurement locations are indicated. An example of the US and NCE-MRA images obtained for one measurement location are shown in the right corner. The section of the NCE-MRA image corresponding to the US image is highlighted.

The total number of consecutive ESRD patients scheduled for vascular access surgery and included in this study was 25 (16 male, 9 female, average age: 68, range 34–84 years). The population of this study is the same as in Bode et al. [10]. A computer model of the circulation, explained in the subsequent section, was personalized for each subject using patient-specific measurements. These patient-specific measurements were conducted as part of the FP7 ARCH study protocol [15], which was approved by the local ethical committee (Maastricht University Medical Center, The Netherlands). Written informed consent was obtained from the patients prior to inclusion. Maximally three months prior to surgery, the patients received both a duplex ultrasound (DUS) and a non contrast-enhanced MRA (NCE-MRA) examination of their arteries and veins to depict the vascular arm geometry (Figure 1). During the decision making for VA surgery, the surgeon had only knowledge of the DUS measurements, not of the NCE-MRA data.

### Computer model

**Figure 2:**
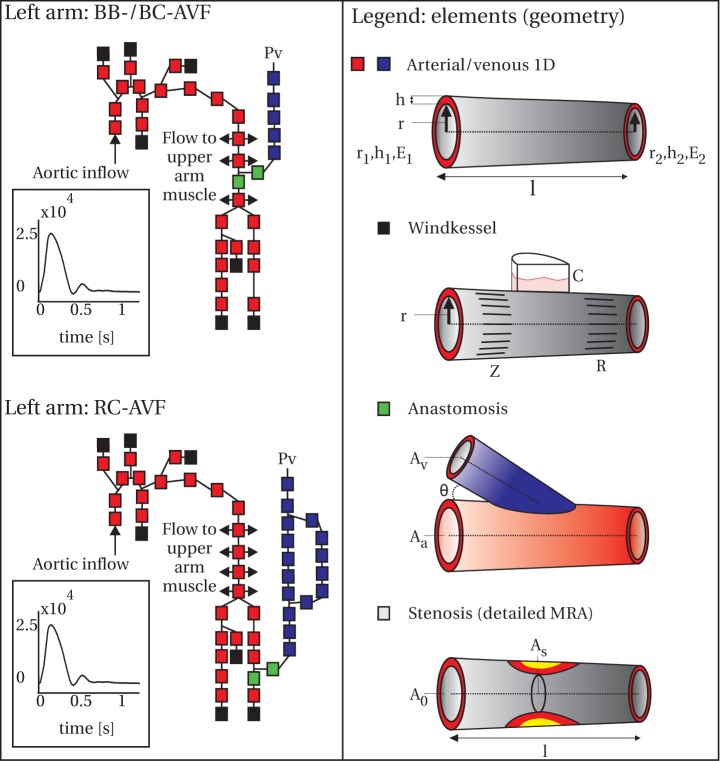
Overview of the computer model geometry and element types. Left: Schematic overview of the upper and lower arm generic geometries, divided into elements for the computer model. Inflow to the circulation was defined by a characteristic flow curve with an average flow of 5100 ml/min. At the end of the venous system (subclavian vein) a pressure of 10 mmHg was prescribed. The right panel shows a legend of the element types in the computer model and their (geometrical) parameters: radius (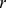), wall thickness (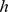), Young's modulus (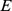), area (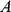), length (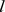), windkessel impedance (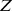, calculated from radius), compliance (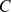), and peripheral resistance (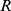). The stenosis element was only used for the cases when the NCE-MRA was studied in detail and a stenosis was present.

In this study, a 1D pulse wave propagation model was used that was originally developed by Bessems et al. [17] and van de Vosse et al. [18], numerically implemented by Kroon et al. [19], and experimentally validated by Huberts et al. [12]. In this model, the vasculature was divided into small (5 cm) 1D elements, for which the local relation between pressure and flow was described by physical laws (1D mass and momentum equations). Each of the 1D elements required specification of the local blood vessel radius (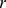), wall thickness (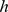), and Young's modulus (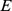) at the proximal and distal end of the element (Figure 2). The intermediate values were obtained by linear interpolation. For the mechanical properties of the blood vessel wall, a constitutive law was used to relate changes in pressure to changes in blood vessel area [19].

The current application focused on the vascular elements between heart and hand. To model the effect of the omitted peripheral blood vessels, a three-element windkessel element [20] (Figure 2) was added at the arterial ends. The preoperative inflow to the circulation was specified by a characteristic aortic flow waveform [21], resulting in a cardiac output of 5100 ml/min. Postoperatively, the cardiac output was iteratively updated until the pressure in the aortic arch was within 5% of the preoperatively measured mean pressure (i.e., baroreflex function).

To model the hemodynamical effects of VA surgery, an anastomosis element (Figure 2) was inserted for the artery-to-vein connection. This element was used to include a pressure drop over the anastomosis that is non-linearly related to flow due to geometrical complexity. The anastomosis element required specification of the area of the feeding artery and draining vein, and specification of the angle between artery and vein. Based on postoperative measurements of anastomosis angle on NCE-MRA data, conducted as part of this study, the VA were modeled with an angle of 45 and 60 degrees was taken for the lower and upper arm VA, respectively.

Since upon detailed inspection of vascular geometry stenoses might be identified in the NCE-MRA data, also a stenosis element (Figure 2) that previously was derived for the 1D pulse wave propagation model [22] was included. The pressure drop due to stenosis geometry is composed of a viscous flow, unsteady flow, quadratic flow, and offset term making the overall effect non-linear. Bessems et al. [22] obtained empirical constants for the stenosis equations using 2D finite element simulations of a 2D parameterized, axially symmetric stenosis geometry. The parameters for this stenosis geometry were the length, and cross-sectional area of the reference and diseased blood vessel (Figure 2).

### Data acquisition

#### Ultrasound

Upper extremity blood vessel mapping by US was already part of the routine preoperative examination in the hospital, which was maximally conducted three months prior to surgery. The extensive blood vessel mapping protocol is in accordance with the highest standards for VA blood vessel mapping in clinical practice [8] and lasted approximately 60 minutes. For full details of the acquisition, see Bode et al. [15]. In short, an ultrasound machine (ALOKA alpha 10) equipped with a 7.5 MHz convex and a 10.0 MHz linear array transducer was used to image the blood vessels of interest. Vascular diameters were assessed at 24 discrete locations along the arteries and veins in the arm (Figure 1). The venous measurements were performed with a tourniquet to enhance diameter reproducibility [23]. After successful acquisition of a cross-sectional image in transversal direction, the blood vessel diameter was measured by a caliper tool. For the veins, the major and minor diameter were noted, while for the circular arteries one diameter measurement sufficed.

Besides the diameter measurements, the inflow to the arm and outflow through the distal arteries was recorded using Doppler US. These flow measurements were repeated directly after surgery, and after 1 week, to monitor the immediate flow increase. The typical duration of these postoperative measurements was 10 minutes. Since the US Doppler method measured the time-dependent maximum velocity in the blood vessel, and the exact velocity profile is unknown, the mean flow was computed as the average of the maximum speed curve, multiplied by the blood vessel cross-sectional area and a velocity profile correction factor of either 0.5 (parabolic) or 1.0 (flat) As a consequence, blood flow measurements are always denoted by a range in this work.

#### NCE-MRA

NCE-MRA was performed in addition to US as novel method for upper extremity blood vessel mapping, to be able to investigate acquisition feasibility in an ESRD population and to study the preoperative upper extremity geometry in detail. The NCE-MRA acquisition protocol has been previously published by Bode et al. [24]. In short, the lower and upper arm were scanned by a 1.5 T MR scanner (Gyroscan Intera, Philips Healhcare, Best, the Netherlands) with the patient in semi-oblique supine position on the arm that was selected for VA surgery. For signal transmission/acquisition a Synergy Flex-L and a Body surface coil (Philips Healhcare, Best, the Netherlands) were used for the lower and upper arm, respectively. A modified version of the steady-state free-precession (SSFP) sequence of Gjesdal et al. [25] was used, because it has reduced sensitivity to phase errors of constant moving spins and turbulent flow compared to other non-contrast-enhanced sequences (e.g. time-of-flight, black blood) [26]. The acquired voxel size was 0.78×0.78×0.79 mm^3^ for all scans and the total acquisition time was approximately 30 minutes, including preparation.

### Model personalization

The computer model requires a large number of input parameter values. It would not have been feasible to measure all parameters for every patient. However, Huberts et al. [27] determined that for a prediction of the arm inflow only a few parameters should preferable be assessed more accurately. The latter include the diameters of the in- and outflow tract. Many more (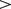
50%) parameters can be chosen generically. Therefore, a generic model of the circulation was used [28], which was personalized by patient-specific measurements. Bode et al. [10] described the personalization based on the US-measurements. Here, a similar approach was followed, but in contrast to Bode et al.[10], personalization was limited to arm geometry (including scaling of non-measurable arteries and veins) and arm flows, which is described in the following sections. As mentioned before, only the arterial geometry could be accurately assessed by NCE-MRA, therefore the venous geometry was personalized based on the US diameter measurements similar to Bode et al. [10].

#### Geometry

The arterial geometry of the arm was personalized with the NCE-MRA data using two levels of detail. First, the vascular geometry was assumed to be linearly tapered per blood vessel, in analogy with the US personalization of Bode et al. [10], thus neglecting stenoses. The cases where flow prediction and postoperative measurement did not overlap were used to study whether an increase in geometrical details improved computer model prediction.

For both approaches, the blood vessels of the VA inflow trajectory were segmented using a semi-automatic centerline detection method, followed by an automatic lumen delineation method [9]. Blood vessel centerlines were automatically determined after provision of a start and end point. These points were the input for a wavefront propagation algorithm [29], which calculated the optimal path of the blood vessel through the 3D NCE-MRA data. Due to the presence of parallel blood vessels and image artifacts, the automatic result had to be verified and corrected to ensure the centerline was representing the blood vessel of interest. Subsequently, the automatic lumen delineation method was applied at discrete positions (each 1 mm) perpendicular to the centerline. The automatic method used the full-width at half-maximum (FWHM) criterion [30], which defines the blood vessel boundary at 50% intensity level between lumen (high signal) and background (low signal). The FWHM-based method was applied along lines at multiple angles through the 2D cross-sectional image of the blood vessel to obtain the local blood vessel diameter. Due to the presence of image artifacts and parallel blood vessels, the diameter measurements were visually verified and incorrect measurements, due to wrong delineation by the segmentation algorithm, were removed prior to further analysis.

**Figure 3:**
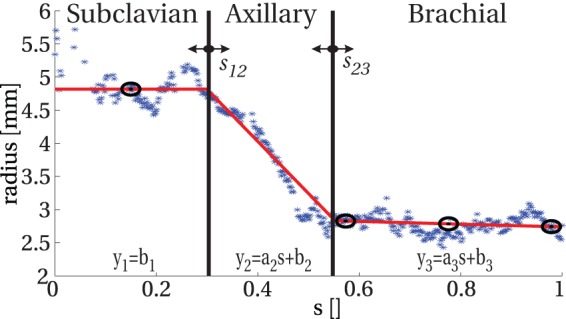
Extraction of subclavian/axillary and brachial artery radius from the original data by three-phase line regression. The blue stars show the original radius values. Variable 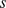 is the normalized position along the curve. The governing equations and the constants to be fitted (parameters: 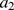, 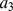, 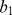, 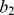 and 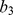, transition points: 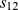 and 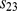) are shown as well. The red line demonstrates the result of the three-phase fitting, while the black circles indicate the fitted values at the vessel mapping locations of the US protocol.

To convert the radius data to patient-specific input for the computer model, a linear fitting procedure was performed for each artery. The radial artery (lower arm) begin and end diameters were computed by a standard least-squares fitting procedure. For the upper arm blood vessels, a different strategy (explained below) had to be employed however, as this part of the circulation contains three arterial blood vessels (i.e., subclavian, axillary and brachial artery) in line with each other, without a clear transition. To generate an accurate linear fit for the three blood vessels, and to estimate the optimal locations of the two transition points, which are a priori unknown, a three-phase line fit was employed [31]. This method performed a least-square fitting procedure of the three individual segments, while the locations of the transitions points were varied. Subsequently, the minimum value of the summed least-squares determined the optimal fit result. To illustrate the three-phase fitting procedure, an example is given for one of the patients in this study (Figure 3). It should be noted that, similar to the personalization approach of Bode et al. [10], a constant value was fitted to the subclavian artery radius profile as large variations in radius were observed in this short segment, which prevented a reliable fit of the derivative.

#### Flow

The computer model was further personalized using the patient-specific flow measure-ments from US. Although flow could be measured by magnetic resonance imaging, ultrasound was used, because flow measurements were already part of the routine blood vessel mapping protocol and postoperative flow was measured by US. The preoperative flow measurements were used to determine the amount of distributed flow to the upper arm muscle tissue, and to determine the outflow at the arterial ends in the lower arm. By subtraction of the outflows (radial/ulnar artery) from the arm inflow (upper brachial artery), the distributed flow was estimated. Subsequently, the windkessel parameters of the arterial ends were calculated using the average blood pressure, average outflow and blood vessel radius [32].

### Monte-Carlo uncertainty analysis

The uncertainty in absolute flow prediction, resulting from uncertainty of computer model input parameters, was calculated by a Monte-Carlo uncertainty analysis. This analysis was purely focused on the effect of geometric input parameters, therefore other model input parameter values were not varied. To perform the uncertainty analysis, each geometric parameter of interest was varied within its uncertainty range, and latin-hypercube sampling [33] was applied to ensure complete coverage of the input parameter space (i.e., parameter interactions were taken into account). A large number of computer model simulations were performed (1000 per parameter) to produce a converged confidence interval of the model output. Only the diameters of the blood vessels in the in- and outflow tract, and a global diameter factor (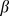), scaling the non-measurable locations of the generic geometry, were varied, because these parameters had the most influence on the arm inflow prediction [27]. In this way, the number of simulations was drastically reduced. The typical number of required simulations for the uncertainty analysis was between 9000 and 15000, depending on the VA configuration, which made the analysis executable on a standard desktop PC (quad core, 3.4 GHz).

The geometric parameter uncertainty ranges, required for conducting the uncertainty analysis, were defined as follows. For each location in the measurement protocol, situated in the VA in- or outflow tract, an uncertainty range was applied whenever a patient-specific value was present. The arterial diameter values were computed from the fitted NCE-MRA data and an uniform uncertainty range of 10% was used, which was in accordance with the maximum expected error due to the segmentation method [34]. The venous diameter values were provided with an uncertainty range of 30%, which attributed for the changes in venous diameter due to day-to-day variations [35]. The parameter variations described here were used for computer model prediction with linearly tapered blood vessels for all patients of the study. For patients for whom an additional investigation of geometry-related error sources was performed, because of non-overlapping results between the computer model and the postoperatively measured flow, additional parameters were added to the uncertainty analysis when a stenosis was identified. Parameter uncertainty ranges of 2 cm, 20%, and 20% were applied on the stenosis length (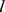), and healthy (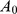) and diseased (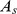) blood vessel area, respectively (Figure 2).

## Results

### Monte-Carlo uncertainty analysis

The comparison of postoperative flow measurements and computer model flow predictions, taking into account the uncertainty of geometric measurements, could be performed for 21 out of 25 patients. For two patients (#8 and #24) a non-standard surgical method was used, therefore these were excluded from further analysis. One patient received a prosthetic PTFE graft (# 15), which was not supported by the computer model. Furthermore, one patient (#21) experienced thrombosis during surgery, which lead to an absence of postoperative flow measurements to compare with. The latter patient was however examined by NCE-MRA prior to surgery, therefore this patient was further analyzed as a patient with non-overlapping flow results.

**Figure 4:**
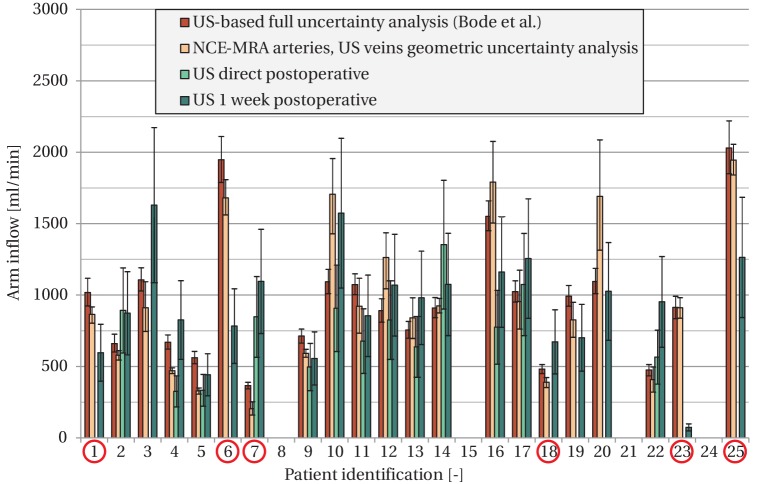
Comparison of postoperative flow measurements (green) with computer model predictions based on geometric uncertainty analysis (orange) and full uncertainty analysis (red, data from Bode et al. [**10**] **).** The error bars of the computer model indicate the 25th–75th percentile interval, while the error bars for the US flow measurements indicate the flow range that results from assuming a parabolic (lower value) or flat (upper value) velocity profile. Patients #8 and #24 were not simulated, because a non-standard surgical method was used. Patient #15 received a prosthetic PTFE graft, which could not be simulated, and patient #21 experienced thrombosis during surgery, which made postoperative flow results unavailable. Cases for which no overlap exists between the geometric uncertainty intervals of the computer model and postoperative flows are indicated with red circles (6/21). These were used for identification of geometry-related sources of error. (a). Patient #23 with thrombosis reported at one week post-surgery. A relevant section of the upper basilic vein is displayed with a significant (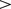
75%) stenosis, indicated by the arrows. (b). Patient #21, who experienced thrombosis during surgery. A relevant section of the lower arm artery (radial) is displayed. Average radius in this section was 0.71 mm, while US reported 1.12 mm. The surgical threshold for lower arm VA creation is at a radius of 1 mm [37].

Figure 4 shows the comparison of the 25%–75% confidence interval of the predicted arm inflow with the postoperatively measured flows (direct/1 week). In this figure, the results achieved in the current study are also compared to those of Bode et al. [10]. It should be noted that the current study used fewer simulations and less parameters in the uncertainty analysis. An overlap in postoperative flow between the clinical measurement and computer model uncertainty intervals was obtained in 15/21 (71%) patients, compared to 16/21 (76%) in the study of Bode et al.[10]. In both studies, the same patients showed overlap, except for patient #18 of the current study. The data of the patients for whom no overlap was found were used for further analysis and these results are the subject of the next section.

### Patients with non-overlapping flows

**Figure 5:**
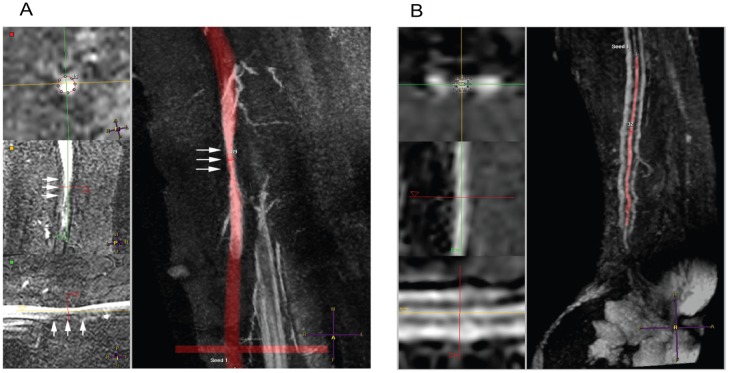
Examples of patients in the non-overlapping group, who experienced thrombosis. The left pane shows orthogonal views on the site of interest, while the right pane displays an overlay between blood vessel segmentation (red) and a maximum intensity projection of the original data. (a). Patient #1, who had a mild narrowing (25%, see arrows) in a 5 cm long section of the blood vessel. The radius profile between start and end is given as well, including the start and end marks of the narrowing. (b). Patient #25, showing a 3.5 cm long severe (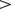
75%) stenosis, indicated by the white arrows. The yellow arrows indicate locations where no valid diameter measurement could be retrieved. These are also indicated in the graph of the radius. Begin and end of the stenosis region are shown in the radius plot as well.

The patient group with non-overlapping flows consisted of 6 patients. Patient #23 reported with thrombosis one week after surgery. Detailed inspection of the NCE-MRA data revealed that this patient suffered from multiple stenoses in the arteries, suggesting peripheral arterial disease (PAD). In addition, the vein that was used for VA surgery (basilic) was stenosed as well (see Figure 5a). These factors could explain the occurrence of thrombosis [36], or at least would indicate an increase in risk. A different case of thrombosis was present in patient #21. Here, the supplying artery in the lower arm (radial) was much smaller on NCE-MRA than reported during US examination. The average radius was 0.71 in the NCE-MRA segmentation, while US reported 1.12 mm. The clinical threshold for a lower arm VA is at a radius of 1.0 mm [37], because a smaller radius is associated with reduced patency [3].

**Figure 6:**
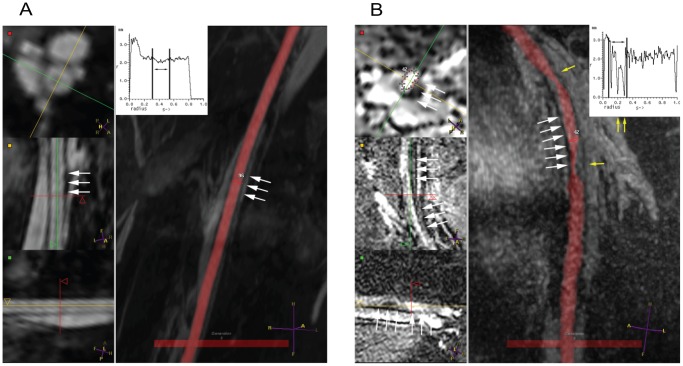
Examples of patients in the non-overlapping group, who have a lower flow postoperative than predicted by the computer model. The left pane shows orthogonal views on the site of interest, while the right pane displays an overlay between blood vessel segmentation (red) and a maximum intensity projection of the original data.

**Figure 7:**
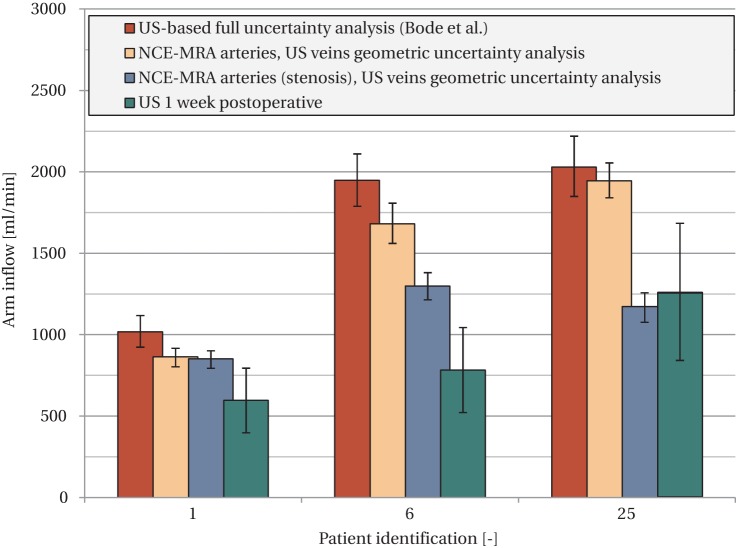
Updated computer model results (in blue) for the three cases identified with stenosis. For comparison the results of the geometric uncertainty analysis without stenosis, and the results of Bode et al. [10] are shown as well. The postoperative US flows are shown in green.

Besides the thrombosis cases, some patients showed lower postoperative flows than predicted by the computer. This triggered further investigation of NCE-MRA data of these patients for potential significant pressure drops in the in- and outflow tract, for example due to stenosis. In three patients (#1, #6, and #25), stenoses were found, of which two are shown here as example cases to demonstrate the effect of a mild and a severe stenosis. Patient #1 had a mild narrowing (25%) of the blood vessel lumen in a 5 cm long section (Figure 6a). Patient #25 had a severe stenosis (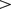
75%) of 3.5 cm long in the arterial inflow tract. For the patients with stenoses, the results with a more detailed computer model, in which the pressure drop due to a stenosis was incorporated, are shown in Figure 7. Two out of the three patients showed an overlap between predicted results and measured postoperative flow upon modeling with increased geometric detail. The other patient remained non overlapping, although the difference with postoperative flow was substantially decreased. Potential secondary (mild) stenosis sites were present in this patient, which might explain any further deviations compared to the postoperative flows.

For the two remaining patients with non-overlapping flow, the deviation from measured postoperative flows could for one patient (#7) be explained by too small US radii (average cephalic radius 0.93 mm) in the outflow tract, compared to the available NCE-MRA information (average radius 2.23 mm). In the other patient (#18) no abnormalities were found in the NCE-MRA data, but the mismatch between predicted and measured flow was small.

## Discussion

Where others [10], [15] have hypothesized that non contrast-enhanced magnetic resonance angiography (NCE-MRA) might provide valuable geometric details for vascular access surgery planning, in this study, these claims were investigated and quantified by means of a computer model study with NCE-MRA based personalization of arterial blood vessel geometry. The advantage of the model-based approach is that the influencing factors, and their interactions are combined by using physical laws, and input uncertainties can be accounted for. Therefore, it eases interpretation of the clinical measurements for VA surgery planning by directly demonstrating the effect on postoperative flow.

The predicted arm inflow of the computer model agreed with the postoperatively measured ultrasound flows in 15/21 (71%) patients, when linearly tapered blood vessels were assumed. These results were comparable to the previous work of Bode et al. [10], despite the fact that in the current study only uncertainty due to geometry was taken into account, which reduced the number of required simulations dramatically. The radius profiles of all blood vessels involved in VA surgery have been studied in detail by applying the automatic FWHM method. Subsequently, for the patients with non-overlapping flows, the present work has searched for geometrical details in the NCE-MRA data, which could explain the difference between computer model prediction and postoperative flow measurements. This detailed exploration of the data revealed the potential cause of thrombosis in two patients. Furthermore, for two out of three patients, identified with stenosis, the computer model flow predictions overlapped with postoperative measurements after taking stenoses into account. The remaining patient potentially had more (mild) stenosis locations. Thus, NCE-MRA is able to spot geometrical details, which provide essential information for the vascular surgeon during VA planning. Moreover, the computer model could use this information to simulate effect of interventions (e.g., balloon angioplasty), which could further support the surgeon.

A limitation of the current methods is that US-based measurements were used to model the venous circulation, because the current NCE-MRA protocol showed a large overestimation (38%, [16]) of diameters, which may be partially explained by the patient position in the MRI. The semi-oblique supine patient position was chosen to optimally benefit from the most homogeneous region of the magnetic field in the MR scanner, since the NCE-MRA is susceptible for artifacts due to magnet inhomogeneity. The bias in diameter measurements is a limitation, which should be resolved prior to incorporating NCE-MRA into clinical routine. Solutions might be an alternative arm positioning, MR scanners with wider bores or high field open MR systems.

Another point of interest for further improvement is the method of postoperative flow determination, because the values obtained with this method were used as reference for clinical validation of the computer model predictions. Here, it should be stressed that the computer model was already validated by in-vitro experiments [12]. For the clinical predictions, the best measurements of direct postoperative flow available under the current study protocol were based on Doppler US. Unfortunately, the measurement uncertainty of these measurements is large due to the required estimation of velocity profile, and due to uncertainty in blood vessel area estimation. Direct postoperative flow measurements could alternatively be obtained by MRI, which is known to have an error in accuracy of only 10% [38]. Upon improvement of these measurements, the computer model could be further optimized and validated.

MRI has the disadvantages of being more expensive and less available than US. Therefore it will probably never completely replace the blood vessel mapping by US. However, based on the results of the current work, it would be beneficial to use NCE-MRA for investigation of patients at risk for peripheral arterial disease (PAD), thrombosis or reduced arm inflows due to local (i.e., stenosis) or global narrowing of the blood vessel lumen. The typical risk factors for blood vessel narrowing would be family history, male sex, age, smoking, hyperlipidema, hypertension, diabetes and prior cardiovascular disease [39]. Furthermore, when the VA choice of an US-based model would differ from the surgeon, NCE-MRA could be used to provide additional information that could alter or confirm the postoperative flow prediction. As the technology is still evolving and the prediction of success will be significantly and additionally improved then the prevention of surgical failure may reduce the actual cost of performing an MRI scan. This technology may become a practical tool for planning and constructing the best access for each patient with reduced complication rates. Therefore, a prospective randomized trial, comparing the surgical outcome using computer model assisted access planning versus regular access planning (including US), is warranted.

In conclusion, the results obtained in this study demonstrate clearly that NCE-MRA does provide valuable geometrical information for planning vascular access surgery. Upon assumption of linear tapered blood vessels an overlap with postoperative flow measurements is obtained in 15/21 (71%) patients. Detailed inspection of the non-overlapping patients revealed that the geometrical details present in the NCE-MRA scans can explain most of the differences, and moreover, when updating the computer model with this information the flow predictions improved. As MRI is more expensive than the traditional blood vessel mapping by US, it would be recommended to examine at least the patients at risk for peripheral artery disease, thrombosis or reduced arm inflow due to local or global narrowing of the blood vessel with this modality as well as patients for whom an US-based model prediction differs from the surgeon's choice.
